# ABO gene editing for the conversion of blood type A to universal type O in Rh_null_ donor‐derived human‐induced pluripotent stem cells

**DOI:** 10.1002/ctm2.1063

**Published:** 2022-10-25

**Authors:** Paolo Petazzi, Laia Miquel‐Serra, Sergio Huertas, Cecilia González, Neus Boto, Eduardo Muñiz‐Diaz, Pablo Menéndez, Ana Sevilla, Núria Nogués

**Affiliations:** ^1^ Josep Carreras Leukemia Research Institute Barcelona Spain; ^2^ Immunohematology Laboratory Barcelona Spain; ^3^ Transfusional medicine. Vall d'Hebron Research Institute (VHIR) Barcelona Spain; ^4^ Department of Medicine Universitat Autònoma de Barcelona (UAB) Barcelona Spain; ^5^ Department of Biomedicine, School of Medicine University of Barcelona Barcelona Spain; ^6^ Centro de Investigación Biomédica en Red de Cáncer‐CIBER‐ONC Instituto de Salud Carlos III Barcelona Spain; ^7^ Red Española de Terapias Avanzadas (TERAV) Instituto de Salud Carlos III (RICORS, RD21/0017/0029); ^8^ Institució Catalana de Recerca i Estudis Avançats (ICREA) Barcelona Spain; ^9^ Department of Cell Biology Physiology and Immunology, Faculty of Biology, University of Barcelona Barcelona Spain; ^10^ Institute of Biomedicine of the University of Barcelona (IBUB) Barcelona Spain

**Keywords:** ABO, CRISPR, gene edition, iPSC, rare blood groups, Rh_null_, transfusion medicine

## Abstract

The limited availability of red cells with extremely rare blood group phenotypes is one of the global challenges in transfusion medicine that has prompted the search for alternative self‐renewable pluripotent cell sources for the in vitro generation of red cells with rare blood group types. One such phenotype is the Rh_null_, which lacks all the Rh antigens on the red cell membrane and represents one of the rarest blood types in the world with only a few active blood donors available worldwide. Rh_null_ red cells are critical for the transfusion of immunized patients carrying the same phenotype, besides its utility in the diagnosis of Rh alloimmunization when a high‐prevalence Rh specificity is suspected in a patient or a pregnant woman. In both scenarios, the potential use of human‐induced pluripotent stem cell (hiPSC)‐derived Rh_null_ red cells is also dependent on ABO compatibility. Here, we present a CRISPR/Cas9‐mediated ABO gene edition strategy for the conversion of blood type A to universal type O, which we have applied to an Rh_null_ donor‐derived hiPSC line, originally carrying blood group A. This work provides a paradigmatic example of an approach potentially applicable to other hiPSC lines derived from rare blood donors not carrying blood type O.

## Graphical abstract

- CRISPR/Cas9 mediated ABO geneedition allows the conversion of blood type A to universal type O in Rh_null_ donor‐derived hiPSCs
- A targeted knock‐in strategyallows to reproduce the c.261delG polymorphism present in the most commoninactive ABO*O.01 allele
- This work provides a newapproach potentially applicable to cell lines established from rare blood donorsnot carrying blood type O

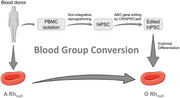

## 1 INTRODUCTION

The transfusion of red blood cells (RBCs), currently obtained from volunteer blood donations, is an essential therapy for patients with chronic or acute anaemia. This form of cell‐based therapy is an indispensable part of modern healthcare systems. However, the perspective of insufficient blood supply due to population aging, and the potential risk of transfusion‐transmitted infections remain major concerns.^1^, ^2^ In addition, the scarcity of donors with rare blood types represents a global challenge when compatible red cells with a rare blood phenotype are required for transfusion.^3^, ^4^ For these reasons, the in vitro generation of RBCs, to supplement the donation system, is nowadays a major focus of research in transfusion medicine.

Beyond transfusion requirements, red cells with infrequent phenotypes are also necessary for diagnostic purposes in clinical laboratories. The identification of unusual red cell antibody specificities in patient sera depends on the availability of reagent red cells with rare phenotypes or infrequent antigen combinations for serological crossmatching, which is crucial to allow the accurate selection and effective search of compatible units for transfusion.

During the past decade, enormous progress has been made in the in vitro manufacture of human RBCs from different cell sources.^5^, ^6^, ^7^, ^8^, ^9^ Among these, human‐induced pluripotent stem cells (hiPSCs) provide an unlimited source of hematopoietic progenitor cells, which can subsequently be differentiated into erythroid cells. hiPSC lines can also be derived from easily accessible peripheral blood mononuclear cells (PBMCs) from selected donors^10^, ^11^ and be amenable to gene editing.^12^, ^13^ Overall, these features make them a promising source for sustainable production of customized red cells.

Different hiPSC lines have already been obtained from existing donors with rare blood types^14^, ^15^ or have been modified using CRISPR/Cas9 gene editing approaches to reproduce uncommon null phenotypes by knocking‐out specific blood group genes.^16^ However, the potential use of hiPSC‐derived red cells is also dependent on the ABO type. Except for the rare Bombay phenotype, extremely infrequent or null blood group types are not necessarily encountered in blood type O donors. Here, we present a CRISPR/Cas9‐mediated ABO gene edition strategy for the conversion of blood type A to universal type O, which we have applied to an Rh_null_ donor‐derived hiPSC line, originally carrying blood group A. This approach is potentially applicable to other hiPSC lines derived from rare blood donors, not carrying blood type O.

## 2 MATERIALS AND METHODS

### 2.1 Ethics approval

Procedures for sample collection and hiPSC line generation were approved by the Clinical Research Ethics Committee (CEIC) of the Vall d'Hebron Research Institute, the *Comisión de Garantías para la Donación y Utilización de Células y Tejidos Humanos* (Spanish National Stem Cell Bank, ISCIII) and the Catalan Authority for Stem Cell Research (0336E/10472/2017). Informed consent was obtained from the donor. All procedures involving animals were approved by the Institutional Animal Ethics Board, and the protocols were approved by the Catalan Authority.

### 2.2 Generation and culture of hiPSCs

To generate Rh null donor‐derived hiPSCs, PBMCs were isolated from a 20 ml‐whole blood sample of the selected blood donor, previously identified and characterized at the Immunohematology Reference Laboratory of the Banc de Sang iTeixits (Barcelona). MNCs were isolated using standard density gradient centrifugation with SepMate™ tubes (StemCell Technologies, Canada). The PBMCs were carefully recovered from the interface and washed in PBS 1×. PBMCs were reprogrammed using the integration‐free CytoTune®‐iPS 2.0 Sendai Reprogramming Kit (ThermoFisher, USA), which contains Sendai virus particles for the expression of the four Yamanaka factors.^17^

Undifferentiated hiPSCs were maintained in mTeSR™1 (StemCell Technologies, Canada) on 3 μg/ml of Laminin‐521 (StemCell Technologies)‐coated plates and expanded using EDTA passaging solution (ThermoFisher). Media samples were routinely tested for the absence of mycoplasma contamination using the selective biochemical test MycoAlert™PLUS (Lonza, Switzerland).

### 2.3 CRISPR/Cas9 gene editing

For *ABO* gene targeting, two strategies were approached: (1) the generation of a gene knock‐out (KO) and (2) the insertion of the naturally occurring c.261delG single nucleotide deletion through a short sequence knock‐in (KI). For both strategies, we designed RNA guides (gRNA) using CRISPR‐direct tool (https://portals.broadinstitute.org/gpp/public/analysis‐tools/sgrna‐design). We selected in both cases the target sites with the lowest number of predicted off‐targets. The gRNA sequences are depicted in Table S1. For the KI strategy, we designed a single‐strand donor DNA carrying the c.261delG mutation and a mutated PAM to avoid re‐cutting of the target sequence (Table S1). Each guide was previously transfected with the Alt‐R® S.p. Cas9 Nuclease V3 (IDT# 1081058) and the Alt‐R® CRISPR‐Cas9 tracrRNA, ATTO™ 550, (IDT #1075927) in HEK‐293T cells according to IDT protocol and tested for cutting efficiency by the T7 endonuclease assay (as described below). The most efficient guide was nucleofected with the Cas9 protein into the parental hiPSC line hiPSC#1 using the Neon Electroporation transfection System (ThermoFisher), and cells were plated into geltrex‐coated plates. Nucleofection efficiency was assessed after 24 h. Forty‐eight hours post nucleofection cells were plated as single cells on geltrex‐coated 96‐well plates for clonal selection in mTeSR™1 (StemCell Technologies, Canada) supplemented with 10 μM Y‐27632 and cloneR (StemCell Technologies). Single‐cell clones were expanded for 2 to 3 weeks and subsequently analysed by Sanger sequencing for the target sites modified.

To check for unintended targets of our sgRNAs, IDT design checker (https://eu.idtdna.com/site/order/designtool/index/CRISPR_SEQUENCE) was used, and the top five in silico‐predicted off‐targets coding regions of ABO E3 sgRNA (CCDC78, HTR5A, PRRG2, RHBDL2, UCKL1‐AS1) and *ABO* E6.1 sgRNA (*C16orf89*, *LINC02794*, *LZTS1*, *NLCN*, *SLC8A1*) were analysed by sanger sequencing. Sequences are available at https://github.com/anasevilla/ABO‐gene‐editing (see Table S2 for sequencing primers).

### 2.4 T7 endonuclease assay

HEK‐293T cultures were dissociated with TrypLE express (ThermoFisher) and 2 × 10^5^ cells were transfected by CRISPRMAX Transfection Reagent (ThermoFisher) with 12 pmol of each of the RNAs (gRNA and tracrRNA) and the Cas9 protein. Genomic DNA was extracted 2 days after transfection. Genomic regions flanking the CRISPR target sites were PCR amplified (Table S1). PCR products were denatured, re‐annealed and subsequently treated with 5U of T7EI at 37°C for 15 min.

### 2.5 *RHAG* and *ABO* gene sequencing

For *RHAG* gene sequence analysis, DNA was extracted from donor PBMCs or hiPSCs by an automated method using the QIAsymphony instrument (Qiagen, Germany). Primers to amplify *RHAG* gene exon 6 are listed in Table S2. For CRISPR edit validation, genomic DNA was isolated from each expanded clone using DNeasy Blood and Tissue Kit (Promega). DNA regions encompassing guide sites were amplified using specific primers (Table S2). Amplification was performed with SequalPrep™ Long PCR Kit with dNTPs (Applied Biosystems, USA). The PCR products were Sanger sequenced using the Big Dye Terminator v1.1 kit (Applied Biosystems). Sequencing primers are also listed in Table S2. DNA sequences were aligned with the reference genomic sequences: NG_011704.1 for the *RHAG* gene and NG_006669.2 for *ABO* gene, using the CLC GenomicWorkbench 21.0.3 software (Qiagen).

### 2.6 Cell line identity

To confirm the cell line identity, genomic DNA was extracted from iPSC clones as well as donor PBMCs and used for short‐tandem repeat (STR) marker analysis using the Mentype® Chimera®system (Biotype®Diagnostic GmbH, Germany).

### 2.7 Karyotype analysis

Genomic integrity of the generated hiPSCs was evaluated by G‐banded metaphase karyotype analysis (Molecular Citogenetics Laboratory, Hospital del Mar, Barcelona). Briefly, cultures of hiPSCs (70% confluent) were treated with KaryoMaxcolcemid (Invitrogen), dissociated, incubated in hypotonic solution and fixed in Carnoy solution (75% methanol, 25% acetic acid). Karyotyping was performed following standard procedures. A minimum of 15 metaphases were examined.

### 2.8 RNA isolation and quantitative reverse‐transcription polymerase chain reaction

Total RNA was isolated from hiPSCs and developing embryoid body (EB) cells using the RNeasy Micro kit (Qiagen) and treated with RNase‐free DNase (Qiagen). Total RNA (1 μg) was reverse transcribed using a high‐capacity reverse transcription kit (Applied Biosystems). All quantitative PCR analyses were performed using the Fast SYBR Green Master Mix (Applied Biosystems) following the manufacturer's protocols on the Light Cycler 480 Real‐Time PCR System (Roche). Gene‐specific primers used for this study are listed in Table S3.

### 2.9 Hematopoietic and erythroid differentiation

hiPSCs were differentiated into hematopoietic progenitor cells (HPCs), using the STEMdiff™Hematopoietic kit (StemCell Technologies) following the manufacturer's recommendations. The 12‐day differentiation protocol was performed in two stages. First, to induce hiPSC commitment towards mesoderm, hiPSC aggregates were plated on laminin‐521‐coated plates and cultured during the first 3 days with STEMdiff™ Hematopoietic *Supplement A* added to the basal medium. Second, for the subsequent 9 days, mesodermal cells were further differentiated into HPCs using basal medium supplemented with STEMdiff™ Hematopoietic *Supplement B* performing half‐medium changes at days 5, 7 and 10 according to the manufacturer's instructions. At day 12, HPCs were harvested from the culture supernatant and re‐cultured in erythroid differentiation media.

To induce erythroid differentiation, HPCs were cultured in basal medium: IMDM (HyClone™, GE Healthcare Life Sciences, USA) containing 1 U/ml Glutamine, 3% (v/v) human AB serum (Banc de Sang i Teixits, Barcelona, Spain), 2% (v/v) foetal bovine serum (Gibco, USA), 10 μg/ml Insulin (Sigma, USA), 3 U/ml heparin (Sigma, USA), 200 μg/ml Iron‐Saturated Transferrin (R&D Systems, USA) and 1U/ml penicillin/streptomycin (Pen/Strep, Gibco) according to the protocol described by Griffiths et al.^18^ In the first differentiation stage (days 0–7), basal medium was supplemented with 10 ng/ml SCF, 1 ng/ml IL‐3 and 3 U/ml EPO (PeproTech, USA); in the second differentiation stage (days 7–11), the basal medium was supplemented with 10 ng/ml SCF and 3 U/ml EPO; in the third differentiation stage (days 11–21), basal medium was supplemented with 3 U/ml EPO and additional transferrin to a final concentration of 500 μg/ml. Cells were cultured into stationary plastic tissue culture flasks at a density of 1–3 × 10^5^ cells/ml at the first stage, 3 × 10^5^ – 1 × 10^6^ cells/ml at the second stage and 1–2 × 10^6^ cells/ml at the third stage by full media changes every other day starting on day 3.

### 2.10 Flow cytometry analysis

Cell surface marker staining was performed by direct immunofluorescence with conjugated monoclonal antibodies listed in Table S4. Briefly, a sample of 1 × 10^5^ cells was incubated with conjugated antibodies in 50 μl of phosphate‐buffered saline (PBS) (Lonza, USA) containing 1% (v/v) bovine serum albumin (BSA) (Grifols, Spain) (PBS‐BSA) for 30 min at room temperature (RT) with continuous mixing in the dark. Cells were washed twice with PBS‐BSA and then analysed on a MACSQuant flow cytometer using MACSQuantify software (Miltenyi Biotech, Germany). For RhAG staining, a sample of 5 × 10^5^ cells was incubated with the monoclonal antibody LA1818 (kindly provided by Prof. Ellen van der Schoot, Sanquin, Netherlands) for 30 min at RT, and then labelled with FITC‐conjugated goat anti‐mouse IgG and IgM (Becton Dickinson) for 30 min at RT in the dark. Results were analysed on a MACSQuant system (Miltenyi Biotech). Cell viability was assessed by 7‐AAD staining.

### 2.11 Preparation of cytospins and May‐Grünwald Giemsa staining

Cytospins were prepared at the Hematological Cytology Service (Hospital del Mar, Barcelona). Briefly, a sample of 1 × 10^4^ cells was prepared by centrifuging onto glass slides at 500 rpm for 10 min in a Thermo Scientific Cytospin 4 cytocentrifuge. The slides were stained with May‐Grünwald Giemsa stain (Merck) according to the Hematology Cytology Service's protocol. Cytospins were imaged at 400× using an optical microscope.

### 2.12 Helix pomatia agglutinin (HPA) Lectin fluorescence staining

Expression of A antigen was analysed by direct fluorescence staining with 50 μg/ml AF488‐conjugated helix pomatia agglutinin (HPA) lectin (ThermoFisher) in living cultured erythroid cells. As positive and negative controls, RBCs from individuals of A and O blood group types were fixed with 4% paraformaldehyde previous direct staining. Nuclei were stained with DAPI. Images were taken using Zeiss Axio Observer Z1 – Apotome inverted fluorescent microscope and analysed using the Image J software.^19^

### 2.13 Serological detection of Rh blood group antigens

Bio‐Rad DiaClon Rh‐Subgroups+K ID‐Cards with monoclonal typing reagents for C (RH2), c (RH4), E (RH3), e (RH5) were used for serological detection of RhCE antigens. Bio‐Rad DiaClon ABD‐Confirmation for Donors ID‐Cards with monoclonal typing reagents for RhD: ESD‐1 M and 175‐2, were used for serological detection of the RhD antigen. Briefly, cell suspensions prepared from 1–2 × 10^6^ hiPSC‐derived reticulocytes were pelleted and resuspended in 50 μl of ID‐Diluent 2 (Bio‐Rad Laboratories, Switzerland). Cards were centrifuged as per the manufacturer's instructions.

A monoclonal anti‐k (Cellano) reagent (Pelikloon IgM monoclonal Lk1) was also used to detect Cellano antigen expression using Bio‐Rad NaCl, Enzyme test and Cold Agglutinins ID‐Cards (Bio‐Rad Laboratories). Fifty microliters of prepared cell suspension were added to a column followed by 25 μl of the anti‐Cellano antibody. Cards were centrifuged as per the manufacturer's instructions.

## 3 RESULTS

### 3.1 Establishment of an Rh_null_ donor‐derived hiPSCs

To obtain an integration‐free Rh_null_ hiPSC line, PBMCs from an Rh_null_ female blood donor were reprogrammed. The donor subject had been previously identified as a homozygous carrier of a single‐base mutation (c.836G > A) in the *RHAG* gene, leading to the rare Rh_null_ blood type (ISBT *RHAG* Blood Group Alleles Table:https://www.isbtweb.org/static/5d593bb0‐02e1‐47a2‐9a8fe3e34df68a5e/ISBT030RHAGbloodgroupallelesv6230‐NOV‐2021.pdf. PBMCs were reprogrammed using integration‐free Sendai virus vectors expressing *OCT4*, *SOX2*, *KLF4* and *cMYC* under serum‐free and feeder‐free conditions.

**FIGURE 1:**
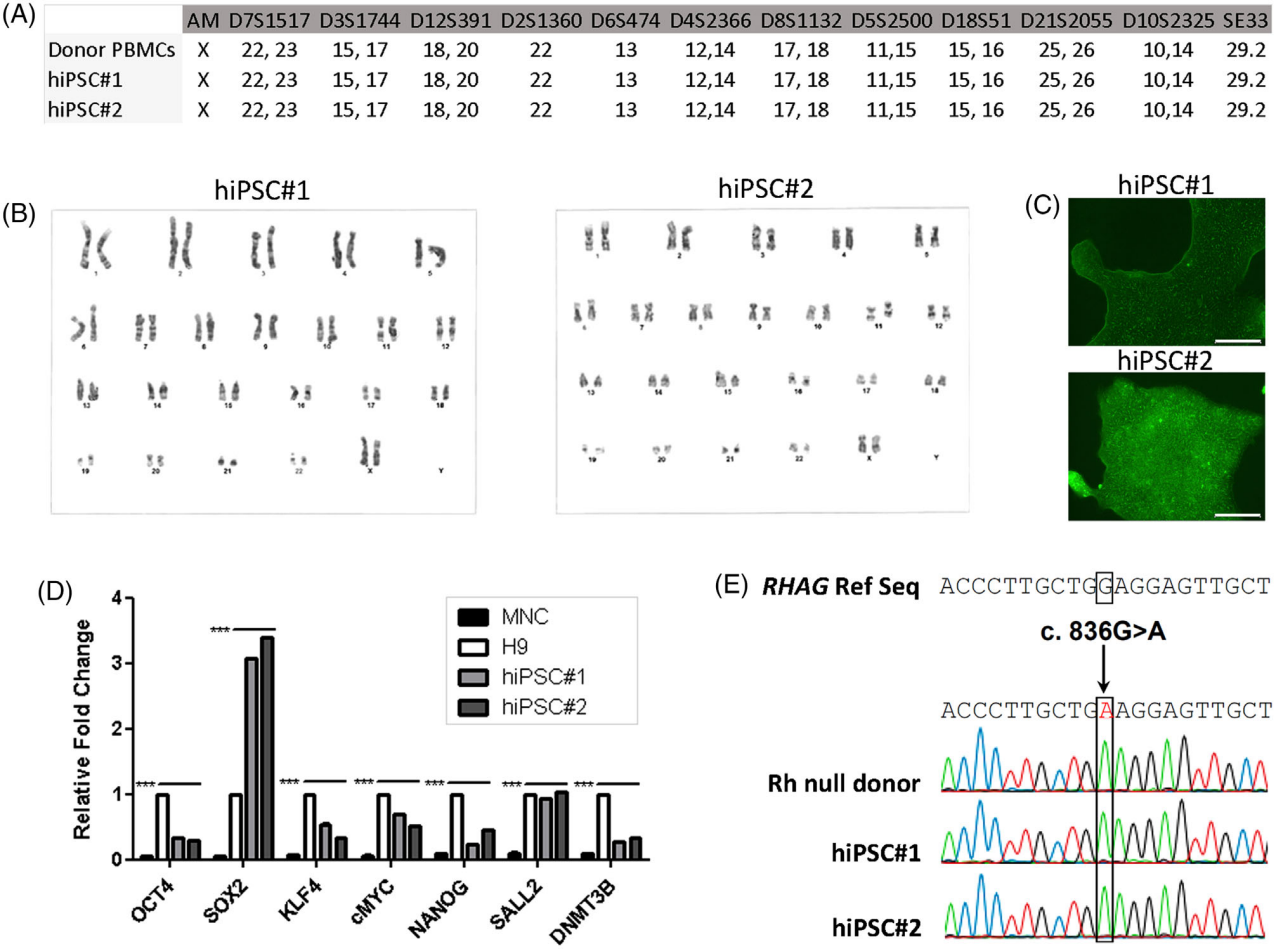
Characterization of the Rh_null_ hiPSC line carrying the c.836G > A mutation in the *RHAG* gene. (A) Short Tandem Repeat (STR) analysis demonstrating identical allelic profiles of the two Rh_null_ hiPSC clones in comparison with the original allelic profile of the donor. (B) Representative karyotype images of the two Rh_null_ hiPSC clones. (C) Representative picture of an AP‐stained hiPSC colony from each clone, captured with an optical microscope. Scale bar 100 μm. (D) Expression levels of endogenous pluripotency genes determined by qPCR, normalized to the *GAPDH* housekeeping gene expression levels. Two‐way ANOVA statistic analysis with Bonferroni posttest, ***p‐value < 0.001. (E) *RHAG* gene exon 6 partial sequence showing the homozygous c.836G > A mutation in genomic DNA from the two Rh_null_ hiPSC clones, as well as in the original donor DNA sample

Two hiPSC lines were generated from this donor and representative clones, named BST PBiPS6‐SV4F‐9 (abbreviated as hiPSC#1) and BST PBiPS6a‐SV4F‐6 (abbreviated as hiPSC#2), were fully characterized. STR analysis confirmed the identity of both lines when compared to the original PBMCs (Figure 1A). Both hiPSC lines robustly proliferated for more than 20 passages, showing a normal diploid female [XX, 46] karyotype, without any detectable numerical or structural chromosomal abnormalities (Figure 1B). The established Rh_null_ hiPSC lines displayed hallmarks of pluripotency, being positive for alkaline phosphatase staining (Figure 1C) and enhanced endogenous gene expression of common pluripotency markers (Figure 1D). The stemness of hiPSCs was also verified by immunofluorescence of pluripotency markers in hiPSC colonies from passages 8 to 15 (Figure S1A). Definitive proof of a pluripotent phenotype was shown in in vitro‐directed differentiation assays towards the three germinal layers (Figure S1B) and in in vivo teratoma formation assays (Figure S1C,D). Additionally, the presence of the *RHAG* gene c.*836G >* *A* homozygous mutation was also confirmed by gene sequencing (Figure 1E). These results demonstrate that we have successfully obtained an integration‐free and feeder‐free hiPSC line carrying the genotype that leads to the Rh_null_ phenotype in derived red cells.

### 3.2 Conversion of blood type A hiPSC line to type O by CRISPR/Cas9‐mediated gene edition

**FIGURE 2:**
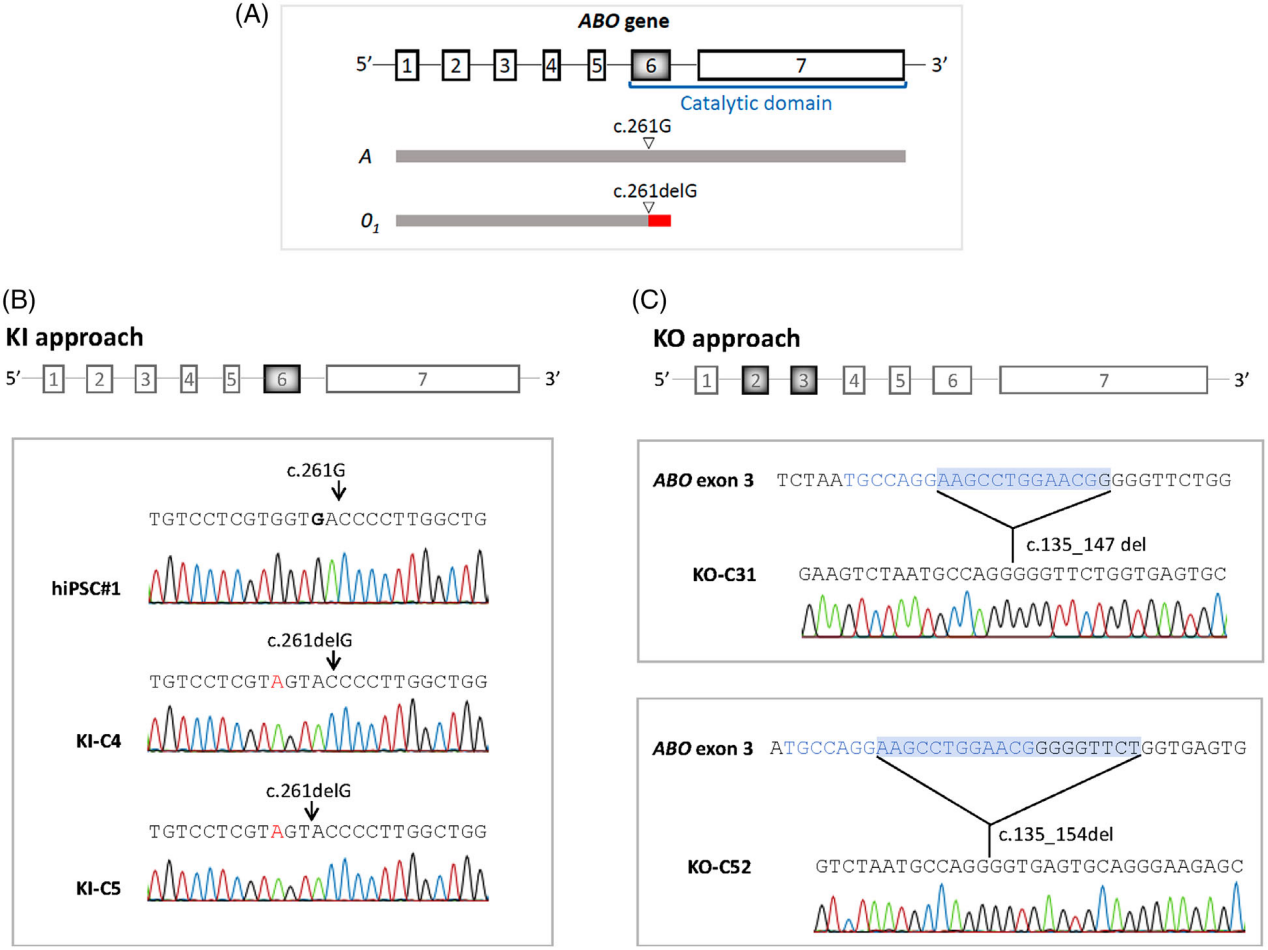
CRISPR/Cas9‐mediated gene edition strategies to convert hiPSCs of blood type A to type O. (A) Structure of the *ABO* gene, located on chromosome 9q34.2. The O allele differs from the A allele by a single nucleotide deletion of guanine (G) at position 261(c.261delG). This deletion leads to a truncated, nonfunctional protein without transferase activity. Adapted from Hosoi et al., 2008^20^. (B) KI approach. *ABO* exon 6 partial sequence confirming homozygosity for the c.261delG deletion in C4 and C5 KI clones. (C) KO approach. Partial sequence showing the region of the *ABO* gene exon 3 containing a 13 bp deletion (c.135‐147del) in KO clone 31 and a 20 bp deletion (c.135‐154del) in KO clone 52. The region matching the RNA guide used to induce the knock‐out is coloured in blue

The original A blood type of the Rh_null_ donor was associated with a heterozygous *A2/O1 ABO* genotype, which was also confirmed in the resultant hiPSC line by *ABO* gene sequencing (Figure S2). In order to convert the Rh_null_ hiPSC line from blood type A to the universal type O, we designed two strategies based on CRISPR/Cas9 technology. The first approach was based on the generation of a KI, mimicking the natural (c.261delG) polymorphism, present in the most common inactive ABO*O.01 (O1) allele. This deletion of guanine in exon 6 causes a frameshift (p.Thr88Profs*31) in a sequence that otherwise is identical to the consensus A sequence (Figure 2A). The second approach relied on the generation of a KO targeting the third exon of the *ABO* gene, also generating a frameshift.

In both approaches, three guide gRNA sequences per CRISPR site were designed, transfected and previously tested on HEK293T cells to evaluate their cutting efficiency through endonuclease T7 assay. In the KI strategy, the best‐performing gRNA, which specifically targeted the exon 6 (Figure 2B), was co‐transfected with the donor DNA sequence containing the 261G deletion, which is used by the DNA repair machinery as the new template after the cut. For the KO generation, the Rh_null_ hiPSC line was transfected by electroporation with the RNP complex whose gRNA targeted the *ABO* gene exon 3 (Figure 2C). Different clones were isolated and screened by Sanger sequencing to confirm the *ABO* gene CRISPR edition. For the KO strategy we checked 26 clones and found five with indels producing a truncated protein (19% efficiency). For the KI strategy, we identified five clones carrying the 261G deletion out of 16 clones screened (31% efficiency) (see data at https://github.com/anasevilla/ABO‐gene‐editing). We then selected two KO (KO‐C31 and KO‐C52) and two KI (KI‐C4 and KI‐C5) clones for further characterization (Figure 2B,C). Using the IDT design checker (https://eu.idtdna.com/site/order/designtool/index/CRISPR_SEQUENCE) software, we analysed the top five in silico‐predicted off‐targets of ABO E3 sgRNA (CCDC78, HTR5A, PRRG2, RHBDL2 and UCKL1‐AS1) and *ABO* E6.1 sgRNA (*C16orf89*, *LINC02794*, *LZTS1*, *NLCN* and *SLC8A1*) by sanger sequencing and found them all consistently unaltered in the four selected clones, demonstrating the specificity of our gene editing strategy (sequences are available at https://github.com/anasevilla/ABO‐gene‐editing).

Importantly, cell line identity of all four clones was confirmed by STR analysis (Figure S3A) and also showed normal diploid [XX, 46] karyotypes (Figure S3B). Furthermore, gene‐edited hiPSC clones remained pluripotent after CRISPR/Cas9 gene editing, retained hESC‐like morphology and expressed similar (p > 0.05) RNA (Figure S3C) and protein (Figure S3D,E) levels of the pluripotency markers SSEA3, SSEA4, TRA‐1‐60 and TRA‐1‐81 to the parental Rh_null_ hiPSC line (Figure S3F). In addition, their pluripotent capacity was tested in vitro through directed differentiation into cell lineages representing all three germ layers through the embryoid body assay (Figure S4A,B). Although expected differences across the iPSC lines were observed for the early lineage differentiation markers (*SOX17, T, TUJ1*) in the differentiating embryoid bodies, no statistical differences were observed for the expression of the pluripotency markers *OCT4*, *SOX2* and *NANOG* at the pluripotency state between gene‐edited iPSC lines and the parental lines (Figure S4C). Thus, we have established two CRISPR/Cas9‐mediated gene edition strategies to convert blood type A hiPSC lines to type O with no impact on the stemness potential.

### 3.3 Morphological changes and immunophenotype confirm consistent erythroid differentiation in ABO‐edited hiPSC lines

**FIGURE 3:**
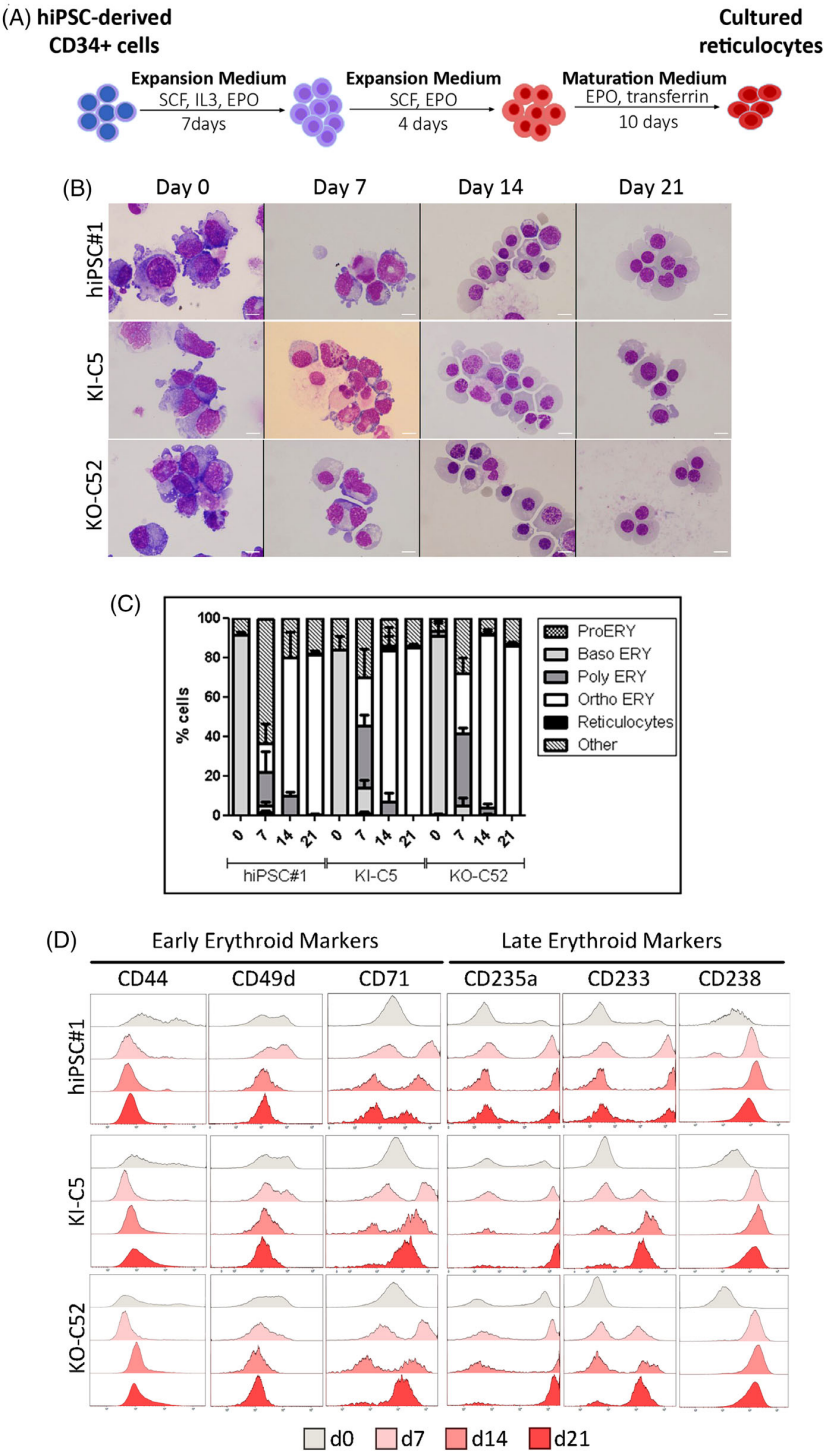
Differentiation of hiPSC‐derived CD34+ cells to the erythroid lineage. (A) Schematic representation of the erythroid differentiation protocol. (B) Representative images of different erythroblast maturation steps from cytospins prepared at days 0, 7, 14 and 21, using May‐Grunwald Giemsa staining. Scale bar: 10 μm. (C) Percentage representation of the different stages of erythroid maturation (pro‐, basophilic, polychromatic and orthrochromatic erythroblasts and reticulocytes) on different days of the erythroid culture (days 0, 7, 14 and 21). (*n* = 3, mean ± SD, Two‐way ANOVA statistics with Bonferroni post‐test, *P*‐value > .05) (D) Flow cytometry analysis of the cell surface markers: CD44, CD49d, CD71, CD235a, CD233 and CD238, during erythroid differentiation at day 0 (grey), day 7 (pink), day 14 (dark pink) and day 21 (red). The ordinate represents the number of cells displaying the fluorescence intensity given by the abscissa Abbreviations: EPO, erythropoietin; ERY, erythroblasts; IL3, interleukin‐3; SCF, stem cell factor.

The potential of the ABO‐edited hiPSC lines to differentiate towards the erythroid lineage was evaluated in parallel with the parental Rh_null_ hiPSCs in three independent experiments. The parental Rh_null_ hiPSCs and the KI‐C5 and KO‐C52‐edited clones were first differentiated towards HPCs with the STEMDiff™ Hematopoietic Kit (Figure S5A). At day 12 of the differentiation protocol, HPCs released from hematopoietic clusters were harvested from the culture supernatant (Figure S5B). This population contained around 90% CD34^+^ cells, and around 60% of these cells were CD45^low/+^ (Figure S5C). To further characterize the CD34^+^CD45^low/+^ fraction, we also analysed the expression of the erythroid lineage surface markers CD71, CD235a, CD49d and CD233. We confirmed the presence of a variable range (36–75%) of early erythroblasts CD71^+^CD235a^+^CD49d^+^CD233^−^ (Figure S5C). The collected cells, containing erythroid‐committed HPCs, were further cultured in erythroid differentiation medium according to the three‐step protocol described in *Materials and Methods* (Figure 3A). The follow‐up of cell viability and expansion throughout the culture showed no statistically significant differences between the edited and the parental cell lines regarding the survival/proliferative capacity of hiPSC‐derived erythroid progenitors (Figure S6A,B). Distinct stages of erythroid maturation were assessed morphologically at four time points (d0, d7, d14 and d21) (Figure 3B). The erythroid cells progressed through distinct erythroid stages with orthochromatic erythroblasts already appearing at day 7, showing no statistically significant differences between the parental Rh_null_ hiPSC and the edited clones, KI‐C5 and KO‐C52, across the 21‐day differentiation period (Figure S6C). At day 21, orthochromatic erythroblasts were the predominant cells (approximately 80%), with a very low proportion of enucleated cells (6–8%), in both the parental Rh_null_ hiPSC line and the edited clones (Figure 3C). Moreover, erythroid differentiation was also assessed by flow cytometry immunophenotyping of erythroid surface markers: CD44, CD49d (α4‐integrin), CD71, CD235a (glycophorin A, GPA), CD233 (Band3) and CD238 (KEL). The observed dynamic changes of expression revealed an analogous progression through erythroid differentiation in parental Rh_null_ hiPSC line and the edited clones. In brief, we observed two distinct patterns of expression (Figure 3D). The adhesion molecules CD44 and CD49d, as well as the transferrin receptor CD71 presented a pattern with high levels of expression in early‐stage erythroblasts and a progressive decrease in late‐stage erythroblasts. In contrast, a different pattern was observed for the CD235a, CD233 and CD238 markers, which displayed low levels of expression in early‐erythroblasts with a progressive increase in late‐stage erythroblasts. Our results concur with the expected progression of erythroid cell differentiation cultures from hiPSC‐derived CD34^+^ HPCs,^21^, ^22^, ^23^ with no significant differences between the parental Rh_null_ hiPSCs and the edited clones (Figure S6D).

### 3.4 The Rh_null_ phenotype is retained in erythroid cells differentiated from parental and ABO‐edited Rh_null_ hiPSCs

**FIGURE 4:**
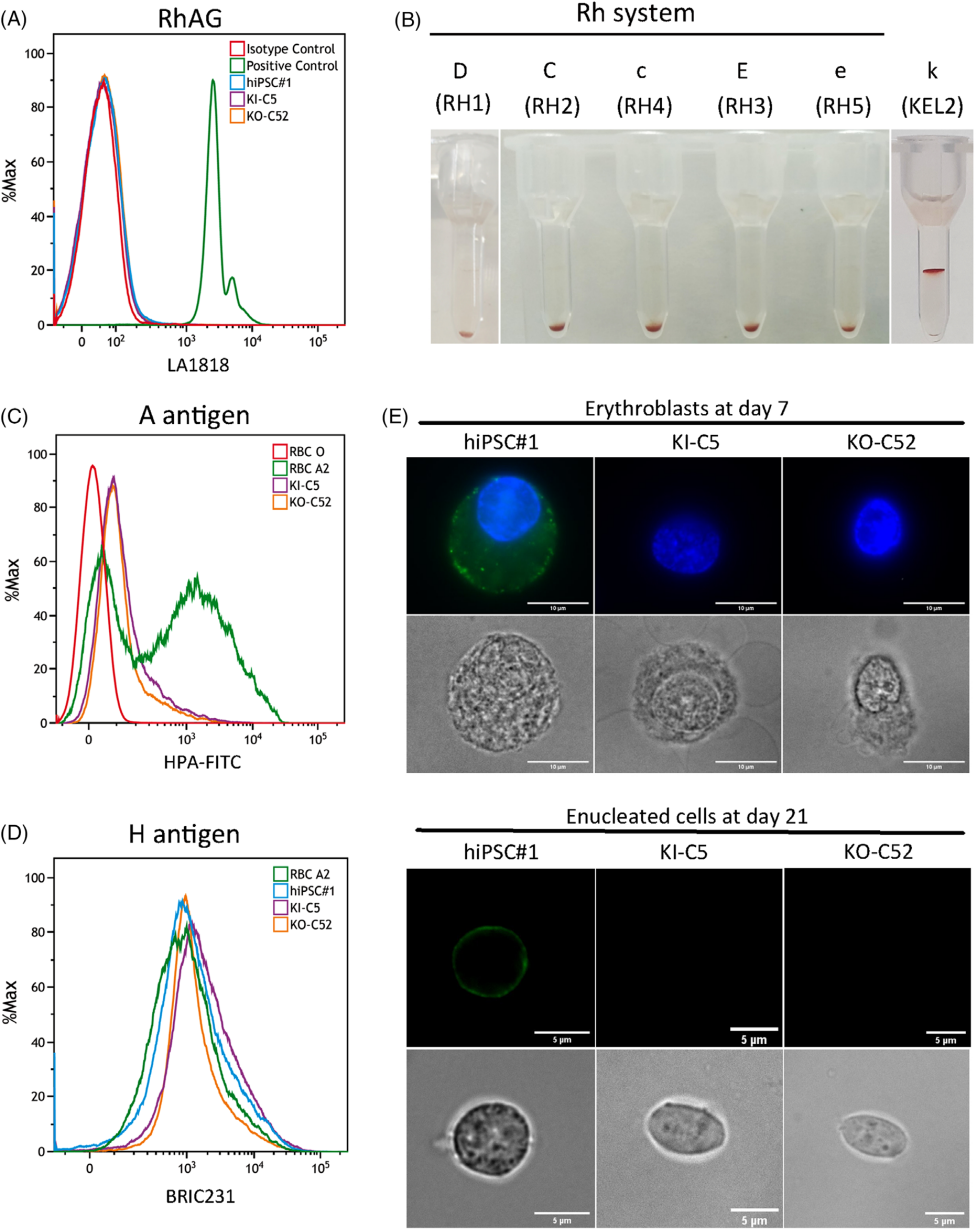
Analysis of the Rh and ABO blood group phenotype. (A) Flow cytometry analysis of RhAG expression in cultured red cells from the parental (hiPSC#1) and edited (KI‐C5 and KO‐C52) lines, at day 21 of erythroid differentiation. RhD+ RBCs were used as a positive control. Mouse IgG1, κ antibody (BD Pharmingen, USA) was used as isotype control. (B) Gel card Rh typing results of cultured red cells from KO‐C52 line showing no agglutination with monoclonal typing reagents for the D (RH1), C (RH2), c (RH4), E (RH3), e (RH5) antigens. Cellano antigen (k) expression is detected in a parallel sample of cultured red cells from the same cell line. (C) Flow cytometry analysis of HPA‐FITC at day 21 of erythroid differentiation. Blood group O RBCs (RBC‐O) and blood group A_2_ RBCs (RBC‐A2) were used as reference. (D) Flow cytometry analysis of H antigen (CD173) at day 21 of erythroid differentiation. Blood group A_2_ RBCs were used as control. (E) Optical microscope images showing specific binding of HPA‐FITC to the cell membrane of differentiated erythroid cells from the parental hiPSC#1 cell line, whereas no labelling was observed in erythroid cells from the edited KI‐C5 and KO‐C52 lines. Nuclei were stained with DAPI. Scale bars: 10 μm (day 7) and 5 μm (day 21)

To confirm the Rh_null_ phenotype, we first assessed RhAG expression by flow cytometry, in cells differentiated from both the parental and ABO‐edited hiPSCs, using the LA1818 anti‐RhAG monoclonal antibody. No RhAG expression was observed on the membrane of erythroid cells derived either from the parental Rh_null_ hiPSCs or from the ABO‐edited clones KI‐C5 and KO‐C52 (Figure 4A). As the RhAG glycoprotein is essential for the Rh complex formation, we next assessed the expression of the Rh antigens (D, C, c, E and e) by agglutination tests using gel card technology, and no agglutination was observed with any of the anti‐Rh typing reagents (Figure 4B), further confirming the Rh_null_ phenotype. These data confirm the successful generation of in vitro differentiated erythroid cells reproducing the Rh_null_ phenotype of the original donor subject, which has neither been affected by the reprogramming of the donor's PBMCs, nor by the CRISPR edition of the *ABO* gene.

### 3.5 ABO blood group conversion in erythroid cells differentiated from ABO‐edited iPSC lines

To analyse A antigen expression in differentiated erythroid cells from parental Rh_null_ hiPSCs and edited clones, fluorescence labelling was performed using HPA, a lectin that has anti‐A human blood group specificity. The erythroid cells derived from ABO‐edited clones were negative for A antigen expression in flow cytometry studies (Figure 4C). Similarly, no differences in H antigen expression were detected between the parental Rh_null_ hiPSCs, expressing blood type A_2_, and the edited lines (blood type O) (Figure 4D). This result indicates that H antigen, which is the precursor for A and B antigen synthesis, is likewise expressed in erythroid cells differentiated from both the Rh_null_ and edited hiPSCs.

In the parental Rh_null_ hiPSC line, we observed early A antigen expression at day 7 of erythroid differentiation and its maintenance until day 21 (Figure 4E). As expected, though, cultured red cells showed weak A antigen expression, in agreement with the original A2 subgroup of the Rh_null_ donor (Figure S7).^24^ In contrast, we could not detect A antigen‐labelled cells in those cultures differentiated from any of the edited clones. These results confirm the successful conversion of blood type A to blood type O using CRISPR‐Cas9 editing strategies in hiPSCs carrying the rare Rh_null_ blood group.

## 4 DISCUSSION

Red cells with rare blood types are currently in limited supply due to the scarce representation of these phenotypes in the global population. The provision of compatible red cells for the transfusion of immunized patients carrying rare blood types is one of the first potential target applications of human red cells manufactured in vitro. On the other hand, the diagnosis of red cell alloimmunization, crucial to ensure the safe transfusion of immunized patients, relies on using carefully selected reagent red cells with well characterized phenotypes. These red cells are also obtained from blood donors, so the availability of infrequent phenotypes needed to properly identify rare antibody specificities is likewise very limited. In this sense, having an alternative (unlimited) cell source to derive red cells with rare blood types in vitro, could potentially overcome the current rare blood limitations in both transfusion and diagnostics.

One of the approaches that have been considered to address this issue, is the use of hiPSCs obtained from existing donors or patients with rare blood types.^25^, ^26^ Of course, such donors are not easily available in practice, as rare phenotypes are usually found in less than 1 per 1,000 in the general population, and donors with exceptional ‘null’ phenotypes are even much less represented. One such example is the H deficiency, also known as the Bombay (O_h_) phenotype, which is found in 1 in 10,000 individuals in India. The generation of hiPSCs from the dermal fibroblasts of a Bombay blood‐type individual^14^ provided the first proof of concept for this approach. More recently, hiPSC lines have been obtained by reprogramming erythroid progenitors from peripheral blood of individuals with the Jr(a−) and D− rare blood types,^15^ demonstrating the feasibility of producing autologous hiPSC‐derived red cells for the transfusion of patients with rare blood groups. However, the potential utility of hiPSC‐derived red cells with null phenotypes or infrequent antigen combinations, extends beyond an autologous use. Such hiPSC lines could provide cultured red cells with difficult‐to‐supply blood types for the transfusion of certain groups of immunized patients (e.g., sickle cell disease patients). Likewise, hiPSC lines could solve the limited availability of reagent‐red cells with rare phenotypes, which are also necessary for the identification of rare RBC antibody specificities.

In this study, we present the generation of a hiPSC line derived from an Rh_null_ donor with blood type A. The donor subject had been previously identified as a homozygous carrier of the c.836G > A single‐base mutation in the *RHAG* gene, leading to the rare Rh_null_ blood type.^27^, ^28^, ^29^ The Rh‐blood group deficiency, or Rh_null_, is an extremely rare phenotype which lacks all the Rh antigens on the red cell membrane. Such valuable blood is necessary for the transfusion support of Rh immunized patients, not only those with Rh_null_ phenotype but also patients with antibodies against any high‐prevalence Rh specificity, for whom compatible blood is always difficult to procure.^3^ Nonetheless, the potential use of hiPSC‐derived red cells for both, transfusion and diagnostics, is also dependent on ABO compatibility. Extremely infrequent or null blood group types are not necessarily encountered in blood type O donors, as it is the case in this blood type A Rh_null_ donor. This circumstance limits the potential use of the hiPSC‐derived red cells due to the naturally occurring ABO hemagglutinins. To overcome this limitation, we considered the conversion of blood type A to universal type O.

The conversion of blood group types A and B to universal type O has been pursued for a long time through approaches based on enzymatic treatment.^30^, ^31^, ^32^ Blood types A and B differ from type O in the presence of an additional sugar residue (GalNAc or Gal, respectively) on the precursor H‐antigen found on type O RBCs. The concept of removing these immunogenic sugars by specific enzymes (glycosidases) from blood type A or B red cells, was first proposed and demonstrated by Goldstein (1982).^33^ The first attempts required massive amounts of enzyme but novel α‐galactosidases and α‐N‐acetylgalactosaminidases have been shown to improve the conversion efficiency.^34^ However, this technology has not yet moved into clinical practice, as there are hold‐ups pending to be solved.^32^

Alternatively, we addressed the conversion of the blood type A Rh_null_ hiPSC line into universal type O using CRISPR/Cas9‐mediated gene editing technology, which allows the precise, robust and efficient edition of genes of interest.^13^ With the aim to abrogate the expression of the α‐1,3‐N‐acetylgalactosamine transferase (A‐transferase), we designed two different *ABO* gene edition approaches. The first approach was based on the generation of a KI, mimicking the c.261delG single nucleotide deletion, present in the most common inactive ABO*O.01 (*O1*) allele.^35^ The specific and precise incorporation of this c.261delG polymorphism within the *ABO* gene has been attempted in the present work aiming to reproduce the genetic basis naturally associated to blood type O. Exploiting the CRISPR/Cas9‐targeted integration to correct genetic defects has led to a number of proof‐of‐principle works in patient‐derived hiPSCs, in which the mutations responsible for cystic fibrosis, haemophilia A and β‐thalassemia were successfully corrected, although with a limited efficiency.^36^, ^37^, ^38^ Thus, a second approach by gene KO was undertaken in parallel to maximize the possibilities to achieve our final goal, which was to obtain an Rh_null_ hiPSC line converted to universal blood type O. Indeed, both the KI and the KO strategies successfully rendered hiPSC‐edited clones with the intended *ABO* gene modifications, as demonstrated by *ABO* sequencing analysis. Moreover, the established *ABO*‐edited hiPSCs lines maintained the Rh_null_‐related *RHAG* gene mutation as well.

The results obtained in the characterization of the KI and KO‐edited hiPSC lines showed no changes on their stemness potential. Likewise, these lines have been successfully differentiated into HPCs and, subsequently, to the erythroid lineage. No remarkable differences have been observed between the parental Rh_null_ hiPSCs line and the edited clones in erythroid differentiation experiments, with overall results concording with the expected progression of erythroid cell differentiation cultures from hiPSC‐derived CD34^+^ progenitor cells.^21^, ^22^, ^23^

It is worth noting that we have been able to produce differentiated erythroid cells reproducing the Rh_null_ phenotype, proving no alteration of the original donor's rare phenotype due to the PBMCs reprograming or to the subsequent hiPSCs CRISPR *ABO*‐gene edition. Remarkably, the results obtained from both KI and KO gene edition strategies provide the first demonstration of blood type A conversion to the universal type O using CRISPR/Cas9 technology. The knock‐out of specific blood group genes, other than *ABO*, in pre‐existing hiPSC lines has been recently reported as an strategy to reproduce uncommon null phenotypes.^16^ Here, we demonstrate the feasibility of robust and sustainable ABO blood type conversion using these newly designed CRISPR/Cas9 gene editing approaches, allowing the production of cultured red cells with improved ABO compatibility. The potential application of these approaches is not restricted to hiPSC lines, since they can also be applied to other cell lines of interest for cultured red cells production, such as immortalized human erythroblast cell lines,^5^, ^39^ derived from individuals not carrying blood type O.

During the past decade, significant advances have been made in the production of manufactured red cells from different cell sources.^7^, ^9^, ^40^, ^41^ Despite the known limitations that still need to be overcome (e.g., low enucleation rate and cost‐efficient scaling), the deeper knowledge of the regulatory pathways involved in terminal erythroid differentiation, together with the continuous progress in scaled‐up protocols and technological achievements, allow to anticipate that in vitro production of RBCs will be possible in the near future. In this context, CRISPR/Cas9‐mediated blood group gene edition will certainly play an important role as a tool to improve blood group compatibility, like this work demonstrates.

## CONFLICTS OF INTEREST

The authors declare no competing interests.

## DATA AVAILABILITY SATATEMENT

The data that support the findings of this study are openly available at https://github.com/anasevilla/ABO‐gene‐editing and in the Supplementary Files.

## Supporting information

Supplement Material

Supplement Material

Supplement Material

Supplement Material

Supplement Material

Supplement Material

Supplement Material

Supplement Material

Supplement Material
