# Supplementary material for: ABO gene editing for the conversion of blood type A to universal type O in Rhnull donor‐derived human‐induced pluripotent stem cells
Source: Clin Transl Med. 2022 Oct 25;12(10):e1063. doi: 10.1002/ctm2.1063 (PMC9593258; doi:10.1002/ctm2.1063)
Supplement: Supplementary file 1 — Supplement Material [file CTM2-12-e1063-s002.docx]

**SUPPLEMENTARY MATERIAL**

**Supplementary Materials and Methods**

***In vitro* differentiation of iPS-derived cells**

Pluripotency was assayed through embryoid body formation (Sevilla et al., 2018). Briefly, hiPS derived cells were dissociated by TrypLE™ Express (Thermo Fisher Scientific) treatment and 40,000 cells were transferred to low attachment 96-well plates (Costar, Corning) in Knockout DMEM supplemented with 20% Knockout serum replacement, non essential amino acids, l-glutamine, penicillin/streptomycin (all from Thermo Fisher Scientific) and b-mercaptoethanol (Sigma-Aldrich) and 10 μM of thiazovivin (Selleck). Cell aggregates (EBs) were cultured for a total of 16 days with media exchanges every other day. EBs were manually harvested at day 16. In parallel, some embryoid bodies were transferred, after 8 days in suspension culture, to gelatin-coated plates and cultured in the same medium for another 8 days for immunostaining of the three lineage markers.

**Immunofluorescence analysis**

Cells or tissues were fixed with 4% paraformaldehyde, blocked and permeabilized with TBS + 0.5% Triton X-100 + 6% donkey serum. Primary antibodies were incubated overnight in TBS+0.1% Triton X-100. Nuclei were stained with DAPI. Confocal images were taken using Leica TSC SPE or SP5 microscopes. Antibodies used are listed in Table S4.

**Teratoma formation**

Severe combined immune-deficient-Beige male mice (n = 2 animal/iPS clone), ∼8 weeks old, were injected with hiPSCs (1 million for each injection site, approximately) subcutaneously in the testicular parenchyma. Mice were sacrificed 12 weeks after the injections or when a tumor was detected by palpation, whichever came first. Teratoma formation was assessed by Hematoxylin-eosin staining and immunofluorescence techniques.

**Supplementary Figure Legends**

**Figure S1. Pluripotency tests of Rh null hiPSC lines. (A)** Confocal images showing immunodetection of pluripotency markers of Rh null hiPSC cell lines. Scale bar: 100 μm. **(B)** Immunofluorescence analysis of *in vitro* differentiation cell cultures using specific antibodies against the endodermal markers FOXA2 and CXCR4, mesodermal markers Brachury and CXCR4, and ectodermal markers PAX6 and TUJ1. Nuclei were stained with DAPI. Scale bar: 100 μm. **(C)** Immunofluorescence analysis of *in vivo* differentiation: hiPSCs were injected into the testis of immunocompromised mice. Teratomas were analyzed by immunofluorescence, showing that the different complex structures were positive for markers of endoderm (FOXA2 and α-fetoprotein), mesoderm (ASMA, ASA) and ectoderm (Neuro200 and GFAP). Scale bar: 50 μm. (**D**) Histological analysis of 12-week teratomas. Identification of bronchial epithelium for iPSC#1 and gland epithelium for iPSC#2 corresponding to the endoderm layer. Presence of cartilage tissue in the teratomas generated by both iPSC lines corresponding to the mesoderm layer. And neural tissue in the teratoma of the iPSC#1 and primitive neuroectoderm tissue for the teratoma generated by the iPSC#2, corresponding to the ectoderm layer.

**Figure S2. *ABO* genotype confirmation in the resultant hiPSC clones. (A)** The *A2* allele differs from the *A1* reference allele in a single cytidine deletion at nucleotide position 1061 that results in an extension of the reading frame by 64 nucleotides. The end of the reading frame is indicated in red. **(B)** Exon 7 of the *ABO* gene was analyzed by Sanger sequencing to confirm the presence of the *c.1061delC* change in the hiPSC#1 clone, characteristic of the *A2* allele.

**Figure S3. Characterization of CRISPR/Cas9-mediated gene edited Rh null hiPSC line. (A)** Short Tandem Repeat (STR) analysis demonstrating identical allelic profiles of each edited KI and KO Rh_null_ hiPSC clones in comparison with the original allelic profile of the donor. **(B)** Representative karyotype images of each characterized edited KI and KO Rh_null_ hiPSC clones. **(C)** Expression levels of endogenous pluripotency genes determined by qPCR, normalized to the *GAPDH* housekeeping gene expression levels (n=3). Two-way ANOVA statistics analysis with Bonferroni post-test, ***p-value<0.001. **(D)** Immunodetection of pluripotency markers using a confocal microscope. Scale bar: 200 μm. **(E)** Analysis of the pluripotent cell surface markers by flow cytometry. **(F)** Analysis of the pluripotent cell surface markers by flow cytometry (n=3) represented as median fluorescent intensity (MFI). One-way ANOVA with Tukey’s multiple comparisons test, *p-value<0.05.

**Figure S4. Characterization of parental and edited lines by EBs differentiation. (A)** Expression of three germ layer markers by immunofluorescence analysis of *in vitro* Embryoid Bodies differentiation cell cultures using specific antibodies against the endodermal marker SOX17, mesodermal marker SMA, and ectodermal marker TUJ1. Nuclei were stained with DAPI. Scale bar: 100 μm; **(B)** Expression levels of pluripotency (*POU5F1, SOX2, NANOG*) and early lineage markers (*SOX17, T, TUJ1*) determined by real-time PCR in the hiPSC lines and in those same lines differentiated to embryoid bodies.**(C)** Expression levels of pluripotency (*POU5F1, SOX2, NANOG*) and early lineage markers (*SOX17, T, TUJ1*) determined by real-time PCR in the parental and edited hiPSC lines. One-way ANOVA with Tukey’s multiple comparisons test, *p-value<0.05.

**Figure S5. Differentiation towards CD34^+^ hematopoietic progenitor cells. (A)** Schematic representation of the hematopoietic differentiation using STEMdiff^TM^ Hematopoietic Kit (StemCell Technologies) which is a two-stage protocol that initially induces cells towards the mesoderm before further differentiating them into hematopoietic progenitor cells. After 12 days, a population of hematopoietic cells containing CD34^+^ CD45^low/+^ progenitor cells can be harvested to further differentiate towards erythroid lineage. **(B)** Representative phase-contrast microscopy images taken at various days of differentiation: day 0, sparse colonies of hiPSCs as small aggregates; day 5, hematopoietic clusters arising from the adherent monolayer; day 12, suspension hematopoietic cells. Scale bar: 200 μm. **(C)** Representative flow cytometry analysis of hematopoietic progenitor cell markers (CD34, CD45) and erythroid-specific lineage markers (CD235a, CD71, CD233 and CD49d) at day 12 of hematopoietic differentiation.

**Figure S6. Erythroid differentiation. (A)** Representative phase-contrast microscopy images taken at days 7 and 14 of erythroid differentiation. Scale bar: 50 μm. **(B)** Cell viability and cumulative expansion from days 0 to 21 (*n*=3, mean±SD; Two-way ANOVA statistics withBonferroni post-test, P-value>0.05). **(C)** Differential counts of stained cytospin samples at days 7, 14 and 21 (hiPSC#1, *n*=3; KI-C5, *n*=3; KO-C52, *n*=3, mean±SD). **(D)** Mean Fluorescence Intensity (MFI) of the erythroid surface markers CD233 and CD235 from days 0 to 21 (*n*=3, mean±SD, Two-way ANOVA statistics with Bonferroni post-test, P-value>0.05).

**Figure S7**.**A antigen expression in blood erythrocytes type A_1_ and A_2_.** Optical microscope images showing specific binding of HPA-FITC to the cell membrane of blood erythrocytes type A_1_ and type A_2_. FITC: fluorescein isothyocyanate; HPA: Helix pomatia agglutinin. Scale bar: 20 μm.

**Supplementary Tables**

| Table S1. List of *g*RNA sequences for CRISPR/Cas9 gene edition | |  |
| --- | --- | --- |
| KO (*Guide 2*, exon 3) | TGCCAGGAAGCCTGGAACGG | |
| KI (*Guide 1*, exon 6) | CAGTAGGAAGGATGTCCTCG | |
| KI donor | CCGCACGCCTCTCTCCATGTGCAGTAGGAAGGATGTCCTCGT  **A**GTACCCCTTGGCTGGCTCCCATTGTCTGGGAGGGCACATTC AACATCGACATCCTC | |
| T7_KO_*Guide 2* Fw | TTGGCAGATGAAGGCCCGTC G | |
| T7_KO_*Guide 2* Rv | GGAGGTCAGGGGTTGAGG TCC | |
| T7_KI_*Guide* 1_ Fw | TGCGTCTCTTGTTTCCTGTCC | |
| T7_KI_*Guide* 1_ Rv | GGTGGGGGAGTTATGTTCCGA | |

| Table S2. Primer sequences for *ABO*, *RHAG* and off-targets gene amplification and sequencing | | |
| --- | --- | --- |
| Locus | **Nucleotide sequence (5'-3')** | **Amplicon size** |
| *RHAG* exon 6 | Fw-TTTCAACTGAAGAATGTGATGTGG* | 320 bp |
|  | Rv-TTTTTCTGCTGGTGGGACAT* |  |
| *ABO*exons 2+3 | Fw-GACCATCTTGGCAGATGAAGG | 1164 bp |
|  | Rv-GAGGTCAAGGCTGACTCCAG* |  |
| *ABO*exons 6+7 | Fw-GGGTTTGTTCCTATCTCTTTGC* | 2273 bp |
|  | Rv-GGGCCTAGGCTTCAGTTACTC |  |
| *ABO*exons 5-6 | Fw-TGCATCCCACGCTTTCCATGC | 831 bp |
|  | Rv-ACTCGCCACTGCCTGGGTCTC* |  |
| *C16orf89* | Fw-tggaccaggtgactggcaga* | 855 bp |
|  | Rv-ctgccactatgtgagacgtccc |  |
| *LINC02794* | Fw-cctgggcaaactttctctctcac* | 896 bp |
|  | Rv-ggagtggatgatggggtgcag |  |
| *LZTS1* | Fw-ggcctggagctggaggtctg* | 837 bp |
|  | Rv-ggttgggatgagaagcagaggg |  |
| *NCLN* | Fw-gagccttttgttccccatgtgg* | 909 bp |
|  | Rv-gccaaagtgagaccccctgg |  |
| *SLC8A1* | Fw-tttctgggtgtccgcgcttcc* | 841 bp |
|  | Rv-cacccctgttctcaactcaacc |  |
| *CCDC78* | Fw-gatgctggagtcactgtctgg* | 750 bp |
|  | Rv-tagtggctgccggtccttgg |  |
| *HTR5A* | Fw-caacaagaactacaacagcgcc* | 886 bp |
|  | Rv-ctggaatatagtccacgcgcg |  |
| *PRRG2* | Fw-tggcattaaccacctgcctgg* | 854 bp |
|  | Rv-ggagggaactcctttagcagcc |  |
| *RHBDL2* | Fw-CAGGCCTGGCTTCAGACTGGA* | 804 bp |
|  | Rv-tggggagaacgtgatggctgg |  |
| *UCKL1AS1* | Fw-aggagctagcccacaggagc* | 881 bp |
|  | Rv-acgctagataagcactgagagc |  |

(*) Primers also used for sequencing

| Table S3. Primer sequences for qRT-PCR | | |
| --- | --- | --- |
| Gene | **Forward (5'-3')** | **Reverse (5'-3')** |
| POU5F1/Oct-4 | TTTTGGTACCCCAGGCTATG | AGAGTGGTGACGGAGACAGG |
| SOX2 | AACCCCAAGATGCACAACTC | GCTTAGCCTCGTCGATGAAC |
| KLF4 | CGAACCCACACAGGTGAGAA | GAGCGGGCGAATTTCCAT |
| cMYC | AGGGTCAAGTTGGACAGTGTCA | TGGTGCATTTTCGGTTGTTG |
| NANOG | ACTGGGAACCTTCCAATGTG | GTGTTCCAGGAGTGGTTGCT |
| SALL2 | TCGCTGAGAAGCCCATCATGAA | TTCCTAGGGTTGGGTCACCAAT |
| DNMT3B | GCTCACAGGGCCCGATACTT | GCAGTCCTGCAGCTCGAGTTTA |
| SOX17 | CGCACGGAATTTGAACAGTA | GGATCAGGGACCTGTCACAC |
| T | ACGCCATGTACTCCTTCCTG | TGAGCTTGTTGGTGAGCTTG |
| TUJ1 | CAGATGTTCGATGCCAAGAA | GGGATCCACTCCACGAAGTA |

| Table S4. Antibodies used for immunocytochemistry (ICC), immunohistochemistry (IHC) and flow cytometry (FACS) | | | | |
| --- | --- | --- | --- | --- |
|  | **Antibody** | **Dilution** | **Catalog number** | **Company** |
| ICC | **OCT4** | 1/500 | 09-0023 | Stemgent |
|  | **NANOG** | 1/100 | 4903 | Cell Signaling Technologies |
|  | **SOX17** |  | AF1924 | R&D Systems |
|  | **TUJ1** | 1/1000 | MMS435P | Covance |
|  | **SMA** | 1/1000 | A2547 | Sigma |
|  | **TRA-1-81** | 1/100 | MAB4381 | Millipore |
|  | **SSEA4** | 1/2 | MC-813-70 | Hybridoma Bank |
|  | **SSEA3** | 1/2 | MC-631 | Hybridoma Bank |
|  | **SOX2** | 1/500 | 09-0024 | Stemgent |
|  | **FOXA2** | 1/50 | AF2400 | R&D Systems |
|  | **CXCR4** | 1/100 | ab124824 | Abcam |
|  | **BRACHYURY** | 1/1000 | AF2085 | R&D Systems |
|  | **PAX6** | 1/100 | PRB-278P | Covance |
| IHC | **Alpha-Fetoprotein** | 1/200 | A0008 | Dako |
|  | **ASMA** | 1/400 | A5228 | Sigma |
|  | **ASA** | 1/400 | A2172 | Sigma |
|  | **Neuro200** | 1/100 | N4142 | Sigma |
|  | **GFAP** | 1/1000 | Z0334 | Dako |
| FACS | **SSEA3** | 1/10 |  | BD Biosciences |
|  | **SSEA4** | 1/10 | 560219 | BD Biosciences |
|  | **TRA-1-60** | 1/10 | 560173 | BD Biosciences |
|  | **TRA-1-81** | 1/250 | 09-0011 | Stemgent |
|  | **CD34** , clone 581 | 1/10 | A21691 | BeckmanCoulter |
|  | **CD45**, clone J.33 | 1/10 | B36294 | BeckmanCoulter |
|  | **CD36**, clone FA6.152 | 1/10 | B43302 | BeckmanCoulter |
|  | **CD235a**, clone 11E4B-7-6 (KC16) | 1/100 | A07792 | BeckmanCoulter |
|  | **CD233**, clone BRIC200 | 1/100 | 9468FI | IBGRL |
|  | **CD71**, clone YDJ1.2.2 | 1/10 | A89323 | BeckmanCoulter |
|  | **CD44**, clone BRIC222 | 1/50 | 9406 | IBGRL |
|  | **CD238**, clone BRIC68 | 1/100 | 9441 | IBGRL |
|  | **CD173**, clone BRIC231 | 1/25 | 9421 | IBGRL |
|  | **RhAG**, clone LA1818 | 1/20 | - | Sanquin |
|  | **Goat Anti-Mouse IgG & IgM** | 1/25 | 555988 | Becton Dickinson |
