# Supplementary figures and images for: ABO gene editing for the conversion of blood type A to universal type O in Rhnull donor‐derived human‐induced pluripotent stem cells
Source: Clin Transl Med. 2022 Oct 25;12(10):e1063. doi: 10.1002/ctm2.1063 (PMC9593258; doi:10.1002/ctm2.1063)

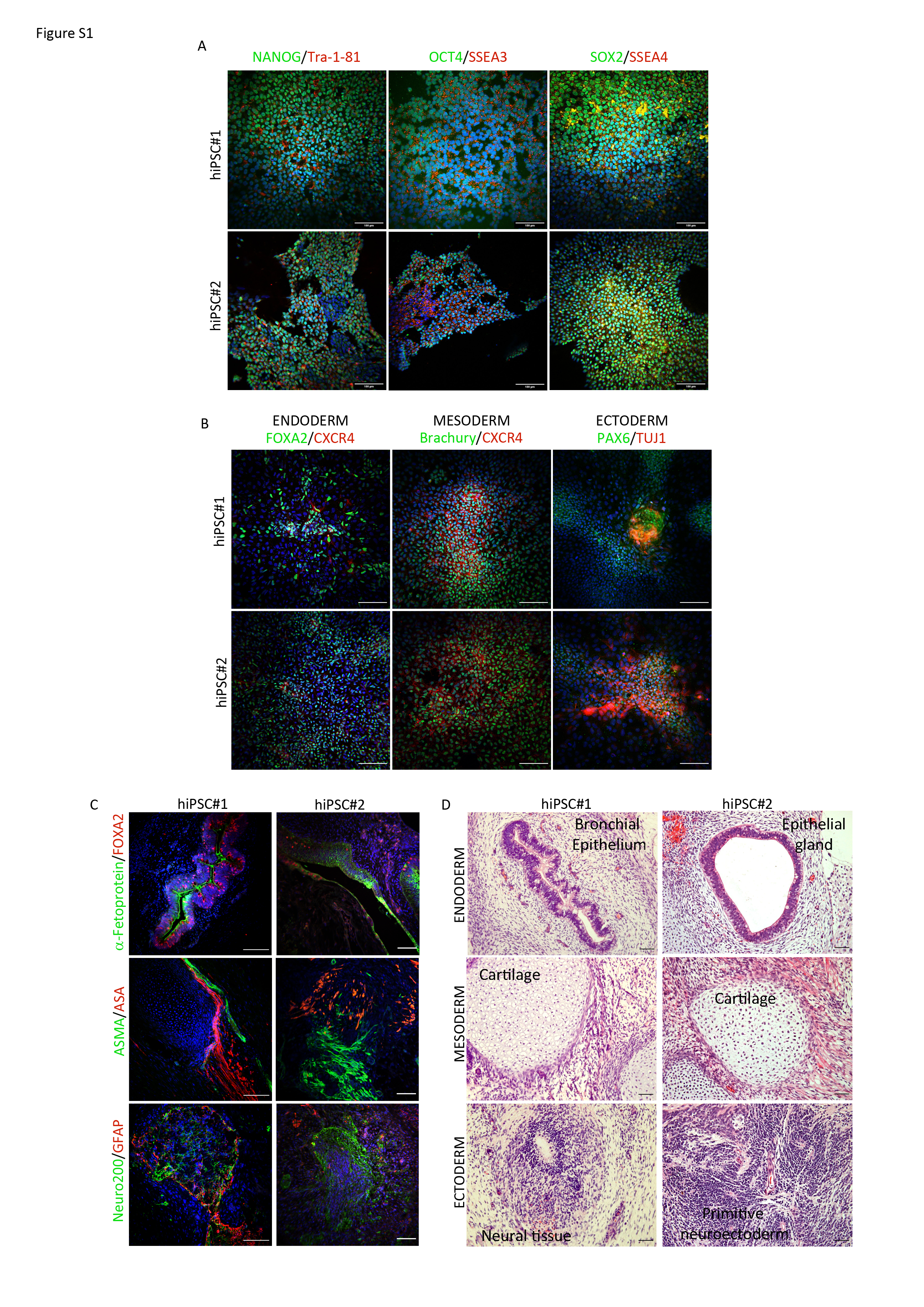

Supplement: Supplementary file 2 — Supplement Material [file CTM2-12-e1063-s004.tiff]

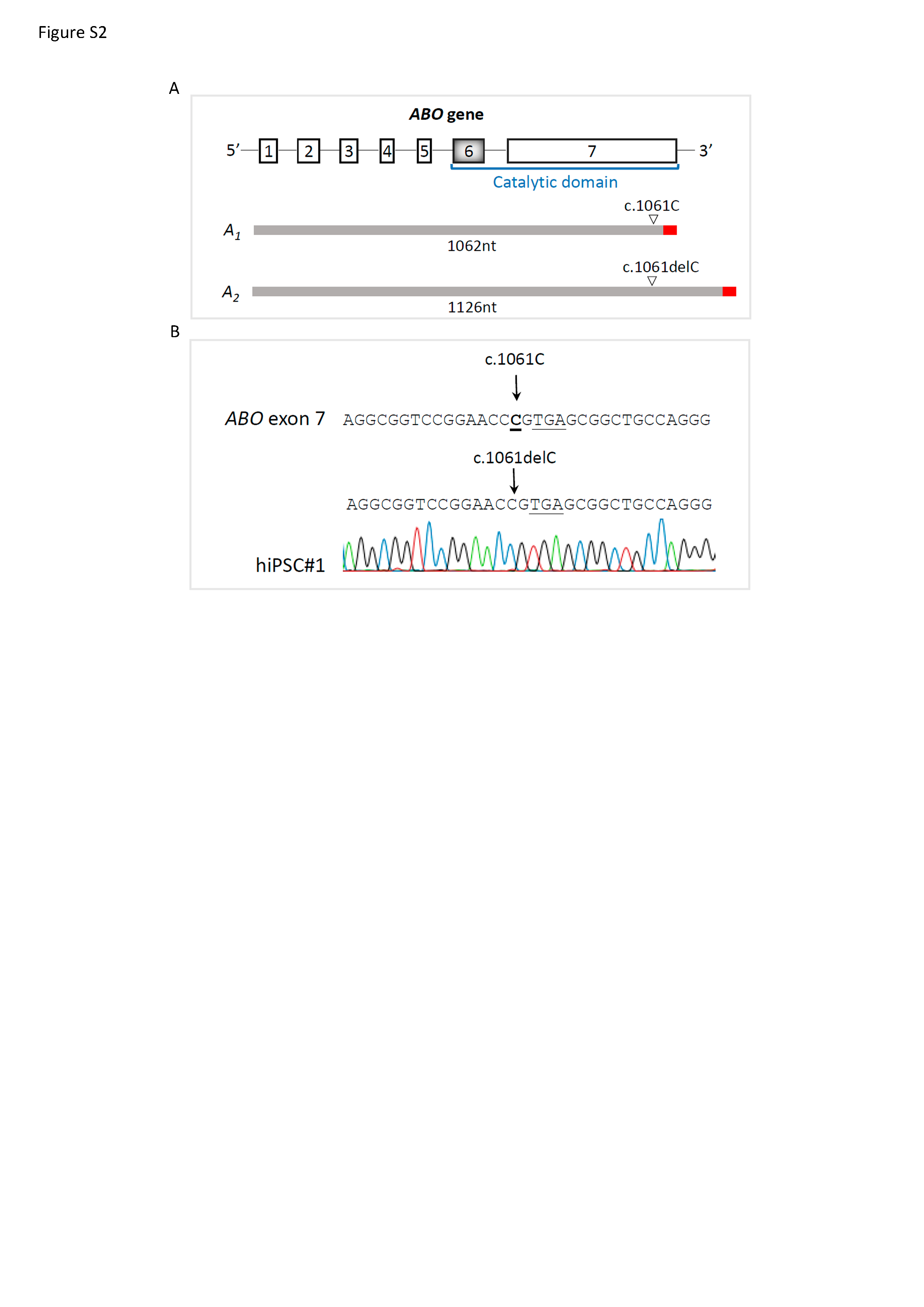

Supplement: Supplementary file 3 — Supplement Material [file CTM2-12-e1063-s008.tiff]

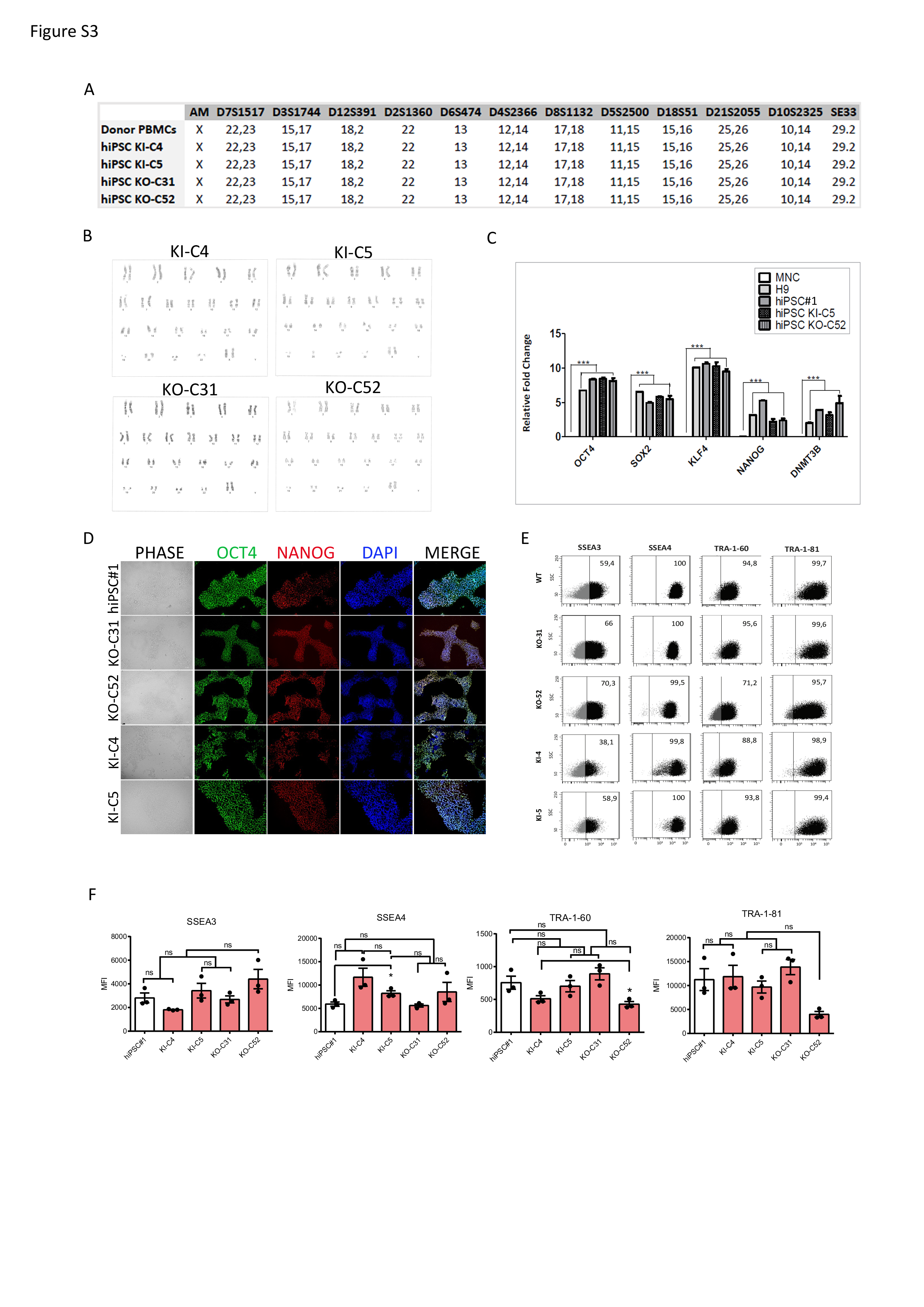

Supplement: Supplementary file 4 — Supplement Material [file CTM2-12-e1063-s001.tiff]

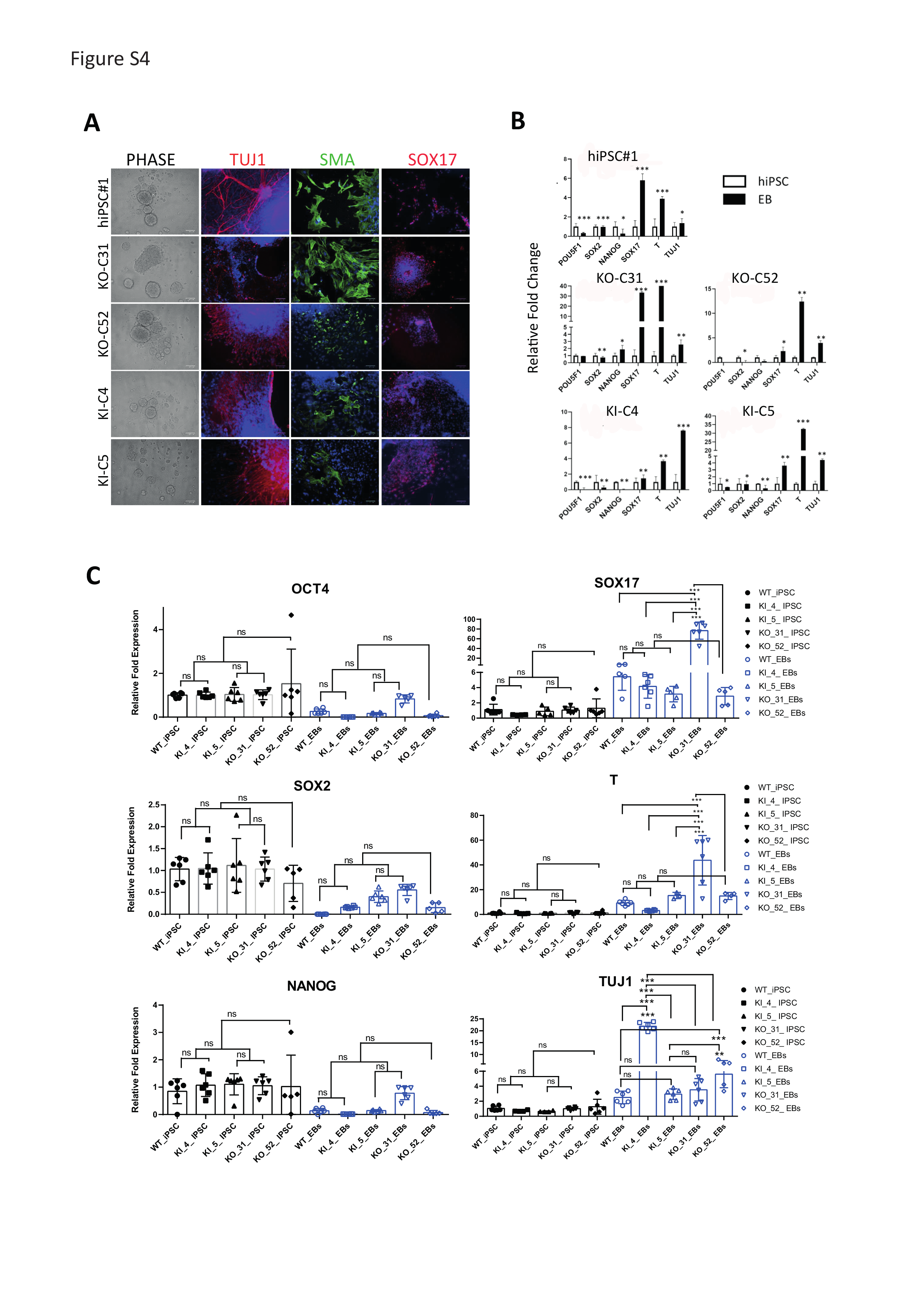

Supplement: Supplementary file 5 — Supplement Material [file CTM2-12-e1063-s003.tiff]

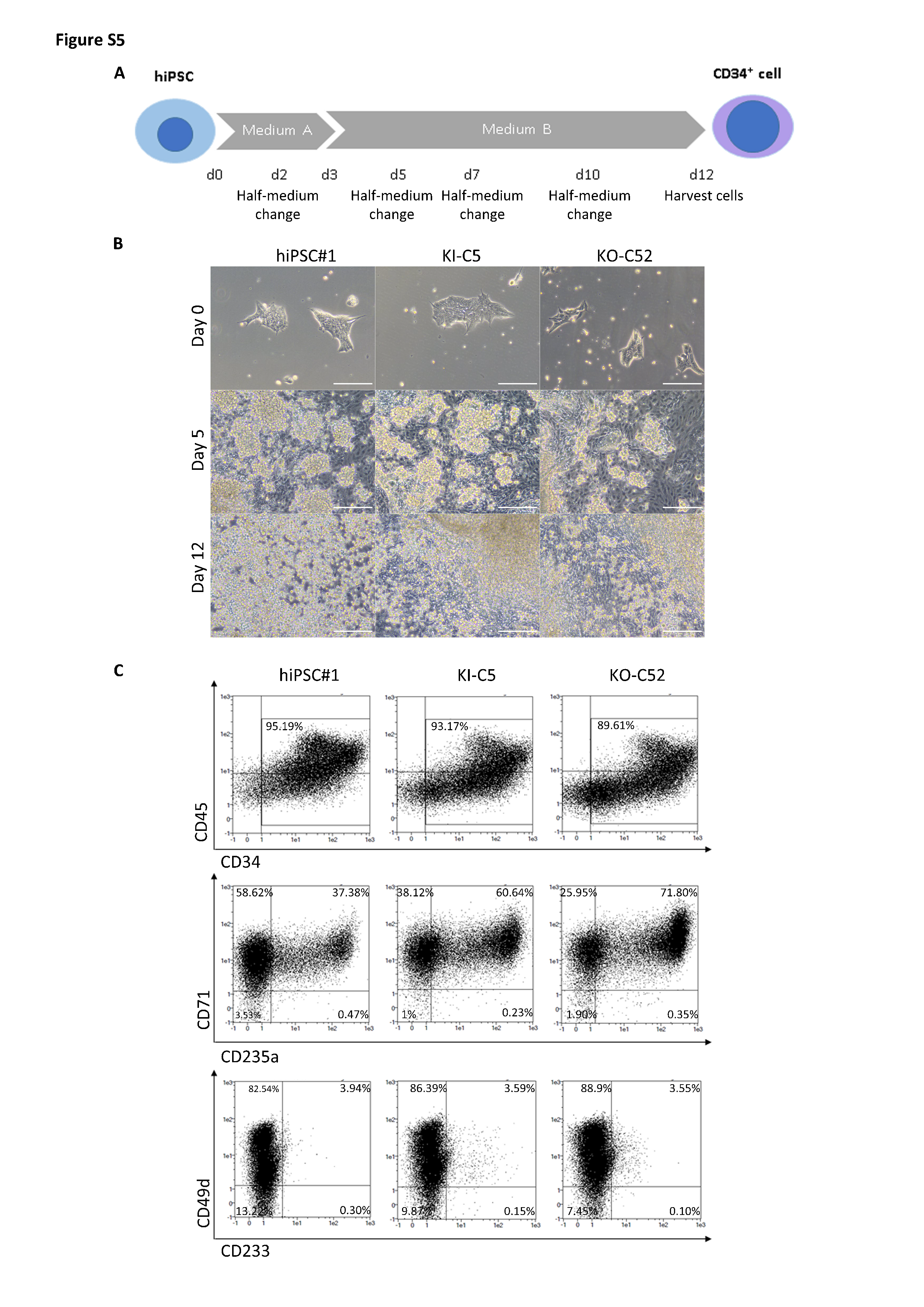

Supplement: Supplementary file 6 — Supplement Material [file CTM2-12-e1063-s005.tiff]

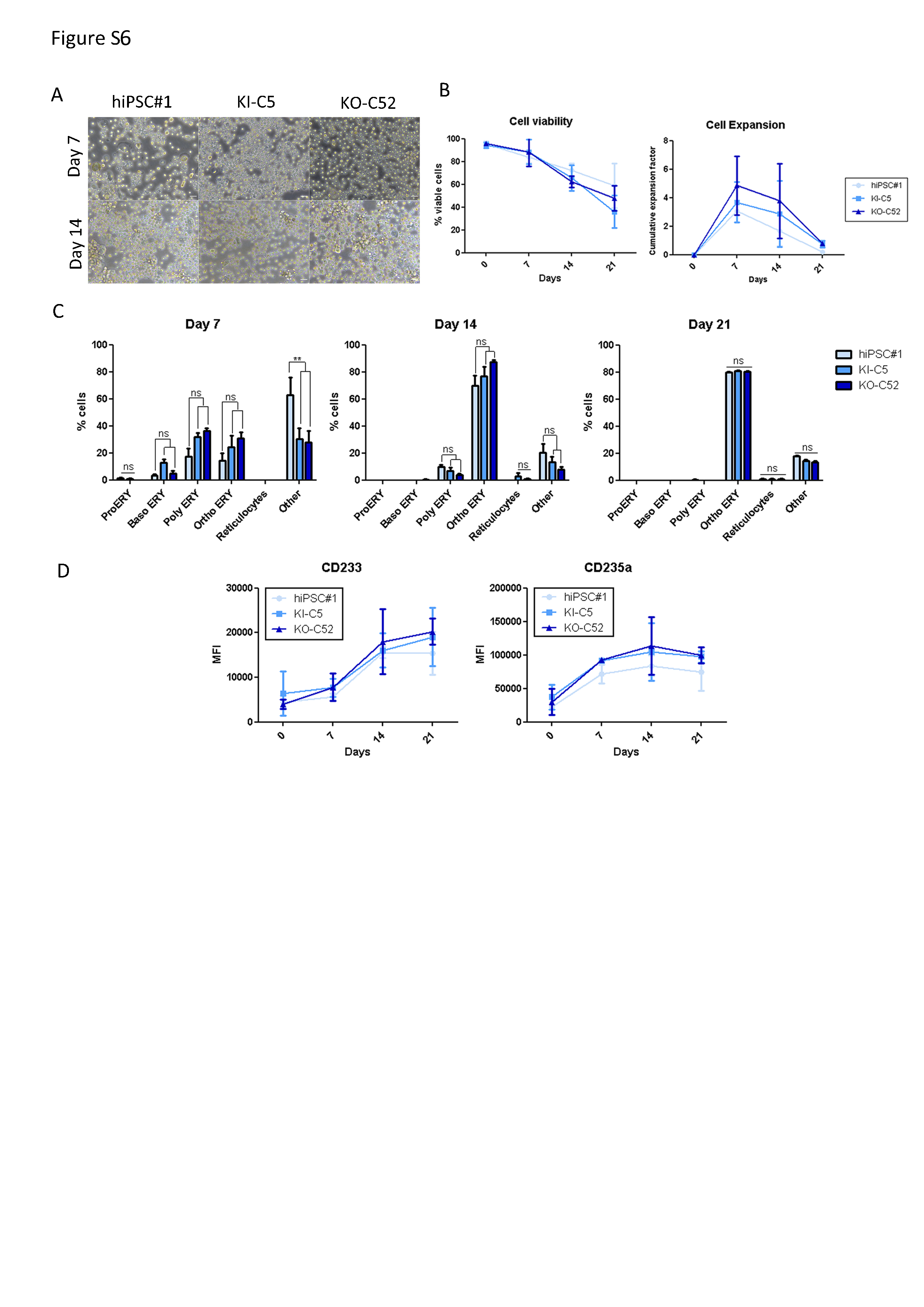

Supplement: Supplementary file 7 — Supplement Material [file CTM2-12-e1063-s007.tiff]

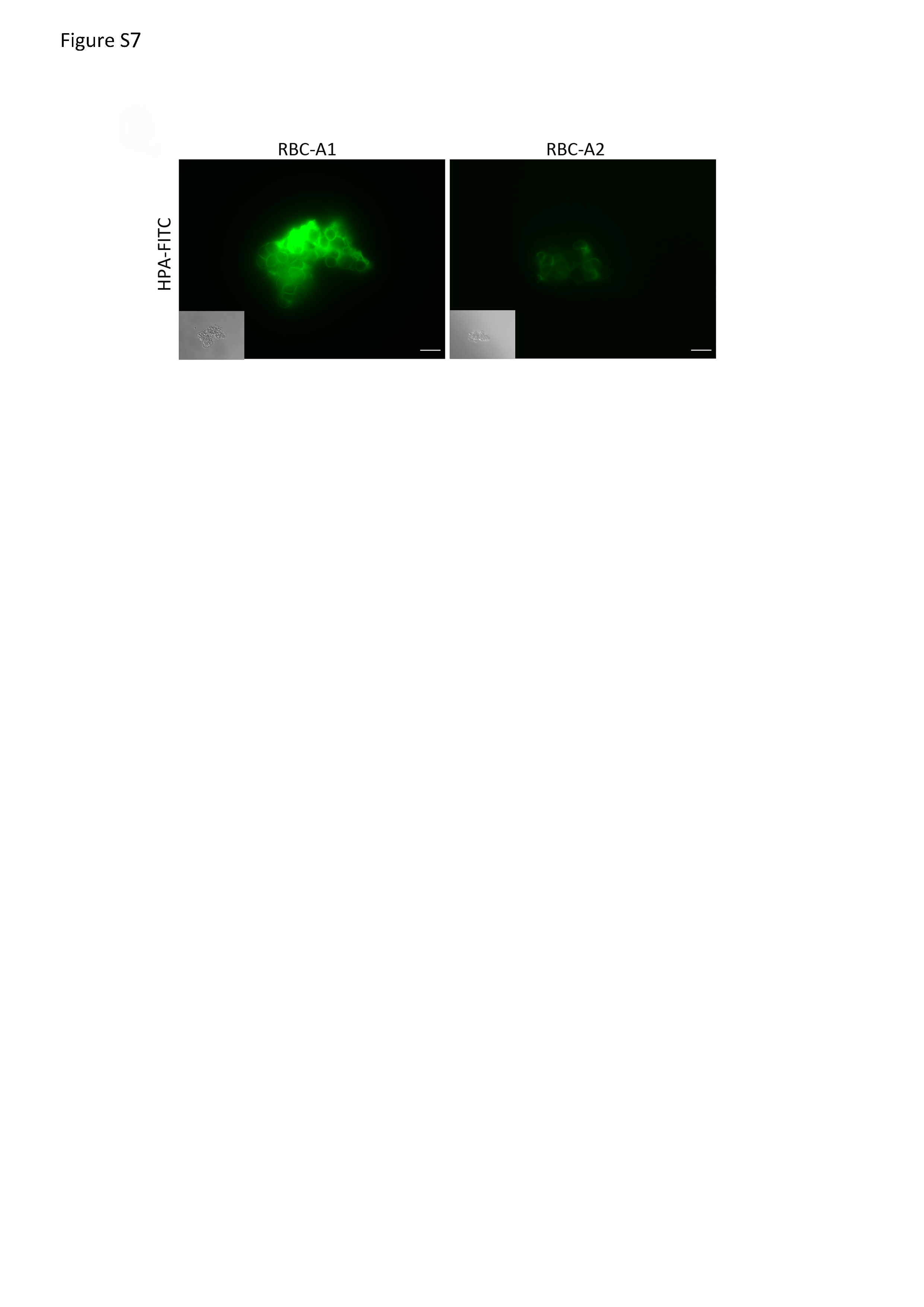

Supplement: Supplementary file 8 — Supplement Material [file CTM2-12-e1063-s009.tiff]

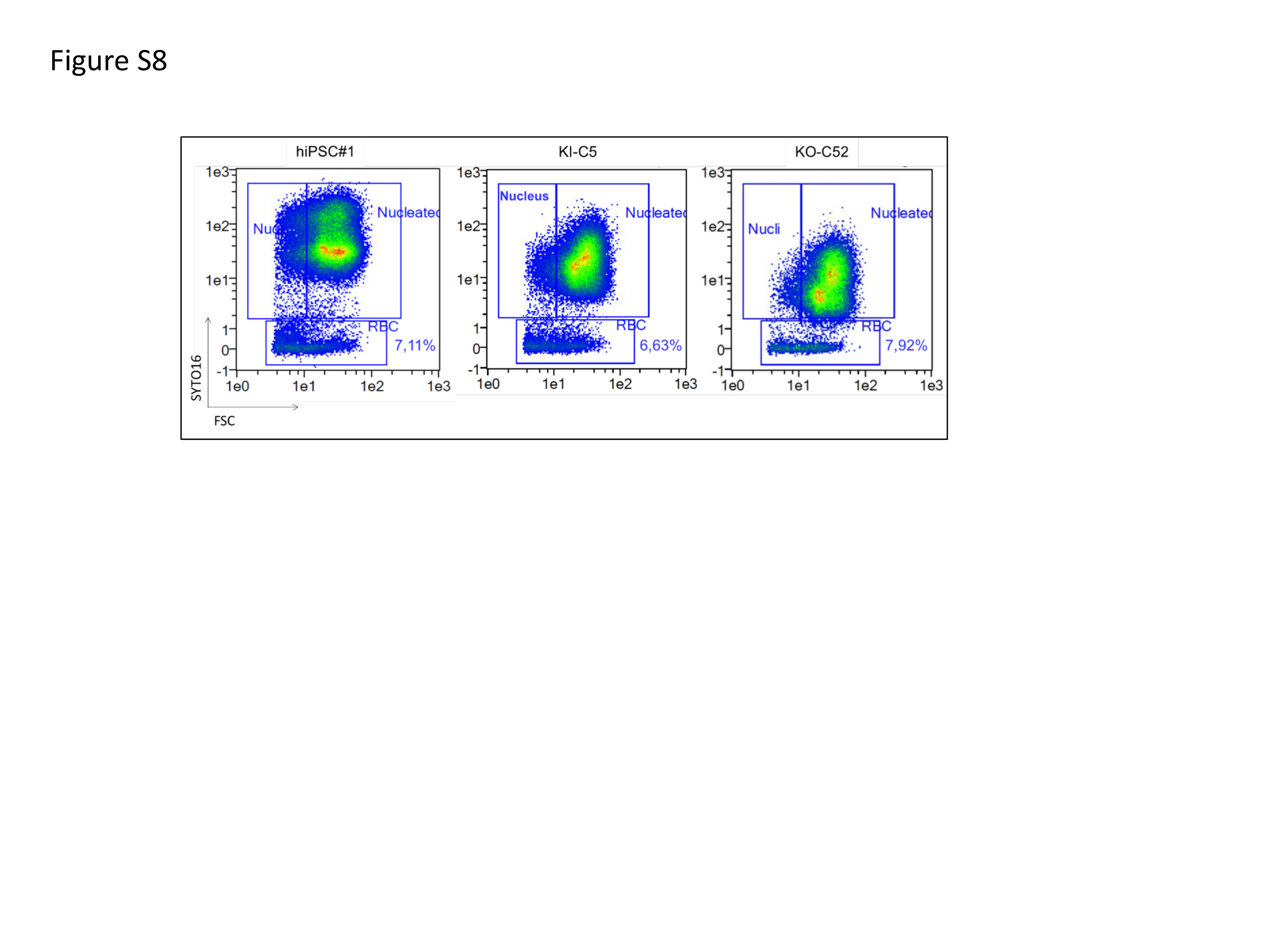

Supplement: Supplementary file 9 — Supplement Material [file CTM2-12-e1063-s006.tiff]
